# Draft Genome Sequence of the Respiration-Competent Strain *Lactobacillus casei* N87

**DOI:** 10.1128/genomeA.00348-16

**Published:** 2016-05-05

**Authors:** Teresa Zotta, Annamaria Ricciardi, Eugenio Parente, Anna Reale, Rocco G. Ianniello, Daniela Bassi

**Affiliations:** a Istituto di Scienze dell’Alimentazione-CNR, Avellino, Italy; b Scuola di Scienze Agrarie, Forestali, Alimentari e Ambientali, Università degli Studi della Basilicata, Potenza, Italy; c Dipartimento di Scienze, Università degli Studi della Basilicata, Potenza, Italy; d Istituto di Microbiologia, Università Cattolica del Sacro Cuore, Cremona, Italy

## Abstract

*Lactobacillus casei* is used as a starter, adjunct, and/or probiotic culture in the production of fermented and functional foods. Here, we report the draft genome sequence of the respiration-competent strain *L. casei* N87, isolated from infant feces. This genome information may be useful for the study of respiratory metabolism in lactic acid bacteria.

## GENOME ANNOUNCEMENT

*Lactobacillus casei* is a versatile lactic acid bacterium (LAB) involved in different food- and health-related applications. Its wide ecological distribution (human host, vegetables, meat and dairy products), high genomic diversity (1–3), and probiotic potential (4, 5) make this species relevant for the production of functional foods and for genetic and physiological studies.

Like other LABs, the members of *L. casei* are generally recognized as oxygen-tolerant anaerobes with fermentative metabolism, lacking both catalase and an active electron transport chain. Recently, Zotta et al. (6) and Ianniello et al. (7) demonstrated that some strains of *L. casei* are capable to grow under aerobic (oxygen) and respiratory (oxygen; hemin and menaquinone in the substrate) conditions, resulting in the expression of phenotypes with enhanced technological properties. *L. casei* N87, isolated from infant feces (8), is a respiration-competent and catalase-positive strain (6, 7); cells growing under respiratory conditions have greater production of biomass and aroma compounds, robustness to oxidative stress, and the capability to remove toxic free radicals.

The whole-genome sequencing of *L. casei* N87 was performed using an Illumina HiSeq 1000 platform (Centre of Functional Genomics, Department of Science and Technology, University of Verona, Italy). The reads were *de novo* assembled using CLC Genomics Workbench version 8.0.3, and the resulting draft genome (average coverage of 586.0×) contained 26 contigs, a circular chromosome of 3,001,027 bp, and an overall G+C content of 47%.

The functional annotation was performed using the NCBI Prokaryotic Genome Automatic Annotation Pipeline (PGAP). A total of 2,671 protein-coding sequences, 79 pseudogenes, 57 tRNA genes, 12 rRNA genes, 1 noncoding RNA (ncRNA), 34 frameshifted genes, and 3 CRISPR arrays were identified in the draft genome of *L. casei* N87.

The genome analysis revealed the presence of genes related to the aerobic (pyruvate oxidase, *pox*; NADH-dependent oxidase, *nox*; NADH-dependent peroxidase, *npr*) and respirative metabolism (cytochrome oxidase operon, *cydABCD*; ubiquinone/menaquinone biosynthesis C-methylase *ubiE*), as well as to oxidative stress tolerance (heme- and manganese-dependent catalases, thioredoxin reductase, glutathione reductase). Superoxide dismutase is lacking in *L. casei* N87.

The draft genome of *L. casei* N87 contains a higher number of CRISPR systems (both CRISPR arrays and CRISPR-associated proteins) compared to other *L. casei* strains (finished, permanent draft, and draft genomes; Integrated Microbial Genomes, https://img.jgi.doe.gov). One of the CRISPR clusters, moreover, is located directly downstream of the *cydABCD* operon. CRISPR systems, belonging to defense mechanism category (V; COGs database), may contribute to the robustness of *L. casei* N87. Eighteen putative horizontally transferred genes from several *Firmicutes* members (IMG platform) are present in the draft genomes of *L. casei* N87.

The genomic information may be useful to confirm the promising features of the respirative strain *L. casei* N87 and to exploit it as a natural boosted culture in different biotechnological applications.

### Nucleotide sequence accession numbers.

This whole-genome shotgun project has been deposited at DDBJ/EMBL/GenBank under the accession number LCUN00000000. The version described in this paper is the first version, LCUN01000000.
